# Diet and Lifestyle Intervention-Induced Pattern of Weight Loss Related to Reduction in Low-Attenuation Coronary Plaque Burden

**DOI:** 10.3390/diagnostics14060615

**Published:** 2024-03-14

**Authors:** Jan Henzel, Mariusz Kruk, Cezary Kępka, Magdalena Makarewicz-Wujec, Łukasz Wardziak, Piotr Trochimiuk, Hubert Krysztofiak, Rafał Dąbrowski, Zofia Dzielińska, Pál Maurovich-Horvat, Marcin Demkow

**Affiliations:** 1 Department of Coronary and Structural Heart Diseases, National Institute of Cardiology, 04-628 Warsaw, Poland; 2 Institute of Pharmaceutical Care, University of Economics and Human Sciences, 01-043 Warsaw, Poland; m.wujec@vizja.pl; 3 Mossakowski Medical Research Institute, Polish Academy of Sciences, 02-093 Warsaw, Poland; 4 National Centre for Sports Medicine, 02-677 Warsaw, Poland; 5 Department of Coronary Artery Disease and Cardiac Rehabilitation, National Institute of Cardiology, 04-628 Warsaw, Poland; 6 Medical Imaging Center, Semmelweis University, 1085 Budapest, Hungary

**Keywords:** coronary artery disease, coronary plaque, coronary computed tomography angiography, DASH diet, lifestyle intervention, obesity

## Abstract

Background: Despite extensive research on body weight and cardiovascular risk, the mechanistic relationship between weight loss and coronary plaque modification has not been adequately addressed. This study aimed to determine the association between body composition dynamics and low-attenuation coronary plaque (LAP) burden. Methods: Eighty-nine participants (40% women, 60 ± 7.7 years) of the Dietary Intervention to Stop Coronary Atherosclerosis in Computed Tomography (DISCO-CT) study with non-obstructive atherosclerosis with nonobstructive atherosclerosis confirmed in computed tomography angiography (CCTA), a randomized (1:1), prospective, single-center study were included into the analysis. Patients were randomly assigned to either experimental arm (intensive diet and lifestyle intervention atop optimal medical therapy, *n* = 45) or control arm (optimal medical therapy alone, n = 44) over 66.8 ± 13.7 weeks. Changes (∆) in body mass (BM) and body composition parameters, including total body fat (TBF), skeletal muscle mass (SMM), and fat-to-muscle ratio (FMR), measured with bioimpedance analyzer were compared with CCTA-measured ∆LAP. Coronary plaque analysis was performed using the 2 × 192 dual-energy scanner (Somatom Force, Siemens, Germany), while quantitative coronary plaque measurements were performed using a semi-automated plaque analysis software system (QAngioCT v3.1.3.13, Medis Medical Imaging Systems, Leiden, The Netherlands). Results: Significant intergroup differences were found for ∆BM (−3.6 ± 4.9 kg in the experimental vs. −1.4 ± 2.9 kg in the control group, *p* = 0.015), ∆TBF (−3.4 ± 4.8% in the experimental vs. 1.1 ± 5.5% in the control arm, *p* < 0.001), ∆SMM (1.9 ± 2.8% in the experimental vs. −0.7 ± 3.2% in the control arm, *p* < 0.001), and FMR [−12.9 (−21.2; −4.3)% in the experimental vs. 3.1 (−5.3; 10.7)% in the control arm, *p* < 0.001]. ∆LAP did not differ significantly between the study arms; however, in the whole study population, ∆LAP was positively correlated with ∆BM, ∆TBF, and ∆FMR (r = 0.45, *p* < 0.001; r = 0.300, *p* = 0.004; r = 0.233, *p* = 0.028, respectively), and negatively with ∆SMM (r = −0.285, *p* = 0.007). Multivariate linear regression analysis revealed the association of ∆LAP with ∆BM, ∆TBF, and ∆FMR. Conclusions: The study intervention resulted in BM reduction characterized by fat loss, skeletal muscle gain, and increased FMR. This weight loss pattern may lead to a reduction in high-risk coronary plaque. Compared to a simple weight control, tracking body composition changes over time can provide valuable information on adverse coronary plaque modification.

## 1. Introduction

Weight control is an important risk modifier for coronary artery disease (CAD). Weight loss is recommended in obese and/or overweight patients with coronary atherosclerosis [1,2]. However, total body mass encompasses several components of distinct biology and metabolism, and it can only be hypothesized that a reduction in different components may have different pathophysiological implications.

Basic research data provide evidence for biological mechanisms through which fat and skeletal muscle may affect atherogenesis. Adipose tissue is capable of synthesizing and releasing a variety of molecules involved in the pathophysiology of inflammation, such as tumor necrosis factor alpha, interleukin 1, interleukin 6, and others [3,4,5,6]. Endothelial dysfunction and inflammation are established precursors of atherosclerosis and thrombosis, directly related to the formation of high-risk coronary plaque and a higher likelihood of cardiovascular events [7]. Skeletal muscle tissue, a substantial part of the fat-free mass compartment, plays an important role in glucose metabolism, which translates into better insulin sensitivity and lower rates of cardiovascular events [8,9,10]. The muscle cells also exert an endocrine role by producing myokines of cardioprotective effects [11]. Observational studies exploring body mass composition in terms of cardiovascular outcomes reported a lower incidence of cardiovascular diseases in subjects with lower fat mass and higher fat-free mass [12,13,14,15,16,17]. The available data suggest independent associations of fat mass and fat-free mass with mortality in the general population [18]. However, the mechanistic relationship between body composition and coronary atherosclerosis has not been adequately addressed. Similarly, no optimal body composition phenotype associated with coronary plaque regression has been identified.

Contrast-enhanced computed tomography angiography (CCTA) is a potent imaging modality that provides insight into the coronary plaque structure and enables the quantification of low-attenuation noncalcified plaque (LAP), which is currently considered a vulnerable plaque component associated with an increased risk of major cardiovascular events [19,20]. Concomitant measurements of LAP and the body components supposedly involved in the process of coronary atherogenesis, i.e., fat, skeletal muscle, and fat-to-muscle ratio, might fill the gap in knowledge regarding the association between weight loss patterns and coronary plaque modification. Based on previously published results from the DISCO-CT study [21], we hypothesized that changes in these body components may affect high-risk coronary plaque modification.

In this context, we aimed to investigate the association between changes in serial measurements of LAP and body composition in a prospectively observed cohort of patients with non-obstructive CAD.

## 2. Materials and Methods

### 2.1. Study Population

The analysis included participants of the Dietary Intervention to Stop Coronary Atherosclerosis (DISCO-CT) study. DISCO-CT was a prospective, randomized, single-center study, in which high-risk plaque reduction was observed among patients subjected to restrictive lifestyle intervention, including the implementation of Dietary Approaches to Stop Hypertension (DASH) diet, atop optimal medical therapy (OMT) compared to patients subjected to OMT alone [21]. The study participants were patients with coronary atherosclerosis confirmed by CCTA, in whom coronary artery stenoses did not exceed 70% and who were qualified to conservative treatment. All participants were subjected to an intensive lifestyle intervention program. As per study protocol, the dietary counselling was provided by a clinical dietitian in a bimonthly basis. Each patient was assigned an individual DASH nutrition plan established after body composition analysis, adjusted to the basal metabolic rate and volume of physical activity. At each energy level (1600, 1800, 2000, or 2600 kcal), the following energy proportions were provided: 52% to 55% from carbohydrates, 16% to 18% from proteins, and 30% from fats. The diet was rich in fruit, vegetables, whole grains, and low-fat dairy products, and restrictive of saturated fats, cholesterol, low-fiber cereal products of high glycemic index, and sweets. Attention was paid to increase the number of meals to 5 per day and to maintain the intervals between meals of less than 3 h.

Apart from dietary intervention, all participants were strongly encouraged to increase physical activity throughout the study. Interview focused on leisure time exercise was completed at each visit and recommendations were given in accordance with the European Society of Cardiology guidelines [1].

We included 89 subjects (40% women, mean age 60 ± 7.7 years) in whom complete data were obtained in serial CCTA studies over the mean observation time of 66.8 ± 13.7 weeks into this analysis. Study participants were recruited from patients in whom CCTA was performed from clinical indications as part of routine CAD diagnostics. In addition to the imaging inclusion criteria mentioned above, the included patients were willing to participate in a lifestyle intervention-oriented program. The main exclusion criteria were type 2 diabetes mellitus (T2DM), cardiomyopathy, heart disease with indications to cardiac surgery within 12 months, genetic familial hypercholesterolemia and/or other congenital metabolic disorders. A complete list of inclusion and exclusion criteria is presented in Appendix A (Table A1). Baseline study population characteristics are presented in Table 1.

**Figure A1:**
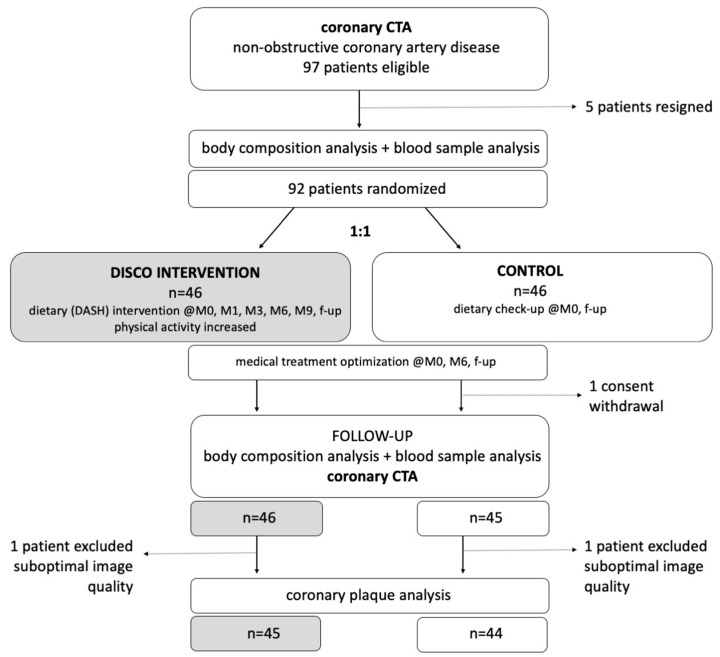
Dietary Intervention to Stop Atherosclerosis in Computed Tomography (DISCO-CT) study design flow chart.

The study protocol and main results can be found elsewhere [5]. Study design flow chart is presented in Appendix B (Figure A1).

### 2.2. Body Composition and Coronary Plaque Measurements

Body composition analysis was performed using InBody S10 bioimpedance analyzer (InBody, Seoul, Republic of Korea). The device is based on multi-frequency bioelectrical impedance analysis technology and uses a 4-pole, 8-point detachable electrode method to measure impedance in five locations at six frequencies (1, 5, 50, 250, 500, 1000 kHz). The results are highly correlated with gold-standard methods [22]. The raw body composition analysis included body mass (BM), body mass index (BMI), total body fat (TBF), body cell mass (BCM), skeletal muscle mass (SMM), and total body water (TBW), expressed in kilograms.

CCTA examinations were performed on a 2 × 192 dual source scanner (Somatom Force, Siemens GmbH, Munich, Germany). Sublingual nitrates (0.8 mg) were administered to all patients prior to the scan. In patients with heart rates > 80/min, intravenous metoprolol was administered (in increments of 5 mg, up to 20 mg). A 50–80 mL bolus of Iomeprol (Iomeron 400, Bracco, Milan, Italy) was injected intravenously at 4.5 mL/s. A retrospectively electrocardiogram-gated acquisition protocol was used, with 192 × 0.6 mm collimation and 70–120 kV tube voltage, which was adjusted according to BMI. Coronary datasets were reconstructed in mid-diastole (60–70% of the R-R interval) and end-systole (40–50% of the R-R interval) with 0.6 mm section thickness and 0.4 mm increment. Image reconstruction was performed routinely using sinogram-affirmed iterative reconstruction (SAFIRE) at a strength level of 3 and the I26f convolution algorithms.

Coronary plaque measurements were performed using QAngioCT version 3.1.3.13, (Medis Medical Imaging Systems, Leiden, The Netherlands), a semi-automated plaque analysis software system based on a two-step (longitudinal and transversal) contour detection approach per vessel for both lumen and vessel contours. The software enables advanced plaque composition analysis according to virtual histology classification based on plaque density. LAP was defined as the coronary plaque of <30 Hounsfield units. LAP burden was calculated by dividing the volume of <30 Hounsfield units attenuation plaque by the vessel volume of the assessed segment, multiplying by 100, and summing on a per patient basis. Imaging data analyses were supervised and approved by a cardiologist experienced in CCTA studies interpretation. Two patients with suboptimal-quality CCTA images were excluded from the analysis (Figure A1).

The changes in the LAP burden, assessed in CCTA, were compared with the changes in body mass (BM) and body mass components. To this end, a three-compartment body composition model was adopted, including total body fat (TBF), body cell mass (BCM), and extracellular mass (ECM), expressed in % of body mass at the corresponding time point of the study. 

Reciprocal changes in body fat and muscle mass were analyzed using the percent fat-to-muscle ratio (FMR), calculated by diving TBF by SMM and multiplying by 100.

### 2.3. Statistical Analysis

Continuous variables were presented as arithmetic mean ± SD and were compared with the Student’s *t*-test when distributed normally; otherwise, median with interquartile range was presented, and the U Mann–Whitney *t*-test was applied. Categorical data were compared using chi-squared or Fisher’s exact test, as appropriate. The *p*-values < 0.05 were assumed significant. To identify possible predictors of LAP regression, multivariate linear regression analysis was employed, including all variables with *p*-values < 0.10 in the univariate regression, and forced changes in the three weight components. Multivariate stepwise regression models were created separately for the change in BM (model A), changes in TBF, BCM, and ECM (model B), and changes in FMR (model C). The *p*-values < 0.05 were assumed significant. Statistical analysis was performed using SPSS software (IBM SPSS Statistics, version 21, IBM Corp., Chicago, IL, USA).

## 3. Results

### 3.1. Baseline Characteristics

The mean baseline BM was 83.6 ± 15.6 kg, and the mean baseline body mass index (BMI) was 29.4 ± 4.0 kg/m^2^. Overall, 78 (88%) patients were overweight (BMI 25 kg/m^2^) and 40 (45%) patients were obese (BMI 30 kg/m^2^). In the whole study group, the mean baseline body mass composition was as follows: TBF 33.1 ± 8.1%, BCM 43.4 ± 5.3%, and ECM 23.5 ± 3.2%. The mean baseline SMM was 37.0 ± 4.8% and the median baseline FMR was 88.5 (65.0; 114.6)%.

The characteristics of the study groups is presented in Table 1.

### 3.2. Body Mass and Composition Profile

**Figure 1:**
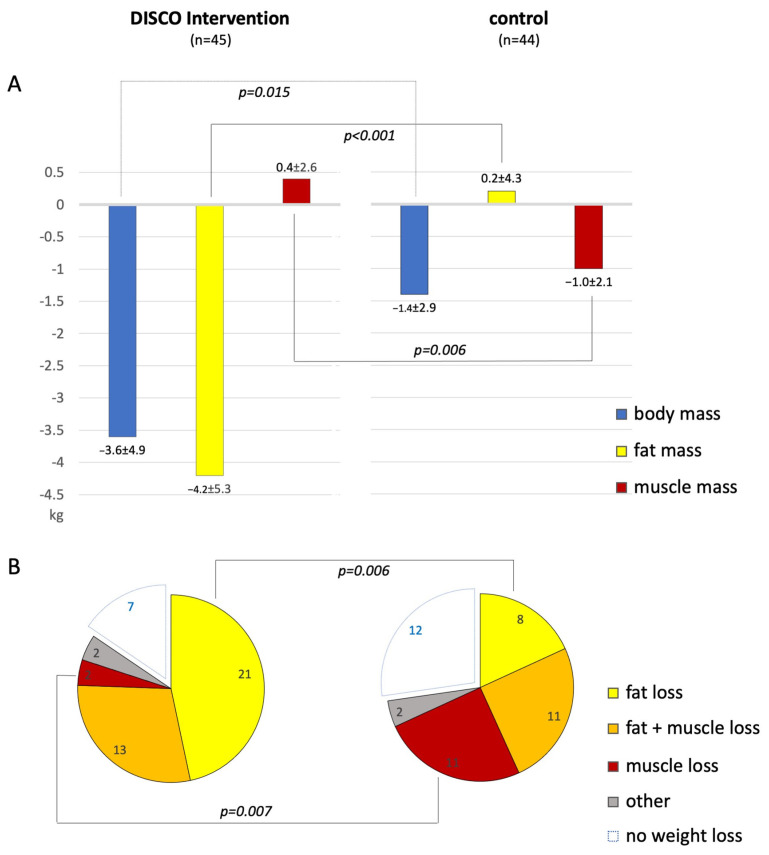
(**A**)—Bar graphs comparing changes in the absolute values of body mass, total body fat, and skeletal muscle mass, all presented in kilograms, in patients subjected to the study intervention (**left**) vs. control (**right**). (**B**)—Pie charts presenting different weight loss patterns in patients subjected to the study intervention (**left**) vs. control (**right**); only *p*-values < 0.05 are presented.

The mean BM did not differ significantly between the study arms either at baseline or at follow-up. However, body mass reduction was significantly higher in the experimental (∆BM = −3.6 ± 4.9 kg) vs. control arm (∆BM = −1.4 ± 2.9 kg; *p* = 0.015); Table 2 and Table 3 and Figure 1A.

Full data on the measured body components are presented in Table 2.

**Figure 2:**
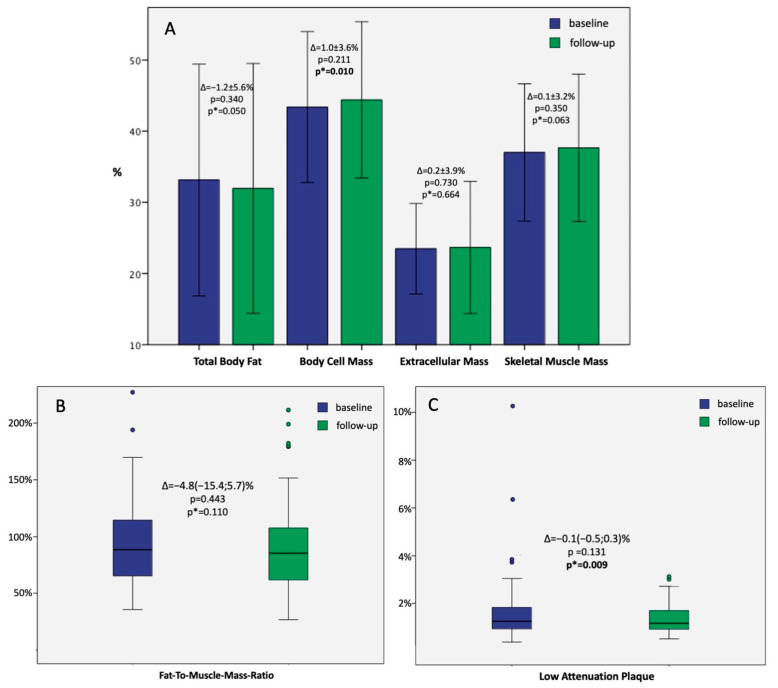
(**A**)—Bar graphs presenting the mean ± 2SD baseline and follow-up values of body components in the whole study population with; those with paired *p*-values are marked as p*. (**B**)—Boxplots presenting the median + IQR of baseline and follow-up fat-to-muscle-mass ratio in the whole study population; paired *p*-value is marked as p*; colored dots represent outliers. (**C**)—Boxplots presenting the median + IQR of baseline and follow-up low-attenuation plaque in the whole study population; paired *p*-value is marked as p*; colored dots represent outliers.

Changes in the absolute values of BM, TBF, and SMM are presented in Figure 1A. Changes in the percent values of TBF, BCM, ECM, SMM, and FMR in the whole study population are presented in Figure 2A,B.

No significant differences were observed between the study groups in the baseline percent body mass components. However, the follow-up TBF was significantly lower in the experimental (30.1 ± 8.0%) vs. control arm (33.9 ± 9.2%; *p* = 0.04). In contrast, the follow-up SMM was significantly higher in the experimental (38.8 ± 4.8%) vs. control arm (36.5 ± 5.3%; *p* = 0.032).

The median follow-up FMR was significantly higher in the control vs. experimental group [93.5 (70.2; 120.2)% vs. 76.9 (56.3; 94.1)%, respectively; *p* = 0.046].

Significant intergroup differences were found for the changes in TBF (−3.4 ± 4.8% in the experimental vs. 1.1 ± 5.5% in the control arm, *p* < 0.001), SMM (1.9 ± 2.8% in the experimental vs. −0.7 ± 3.2% in the control arm, *p* < 0.001), and FMR [−12.9(−21.2;−4.3)% in the experimental vs. 3.1(−5.3;10.7)% in the control arm, *p* < 0.001]. Full data on the percent body mass components are presented in Table 3.

### 3.3. Weight Loss Patterns

Overall, seventy patients (79%) managed to lose weight, 38 in the experimental arm and 32 in the control arm (*p* = 0.2). Fat reduction was observed in 50 (56%) patients and occurred more frequently in the experimental vs. control arm (38 vs. 22 patients, respectively; *p* < 0.001). Skeletal muscle loss was observed in 44 (49%) patients and occurred more frequently in the control vs. experimental arm (28 vs. 16 patients, respectively; *p* = 0.01). Among the weight reducers, pure fat reduction prevailed in the experimental arm (55% of patients), while in the control arm, skeletal muscle reduction was most common (34% of patients, Figure 1B).

### 3.4. LAP Burden Dynamics

In the whole study population, the median LAP burden was 1.25 (0.91; 1.84)% at baseline and 1.16 (0.91; 1.70)% at follow-up (*p* = 0.131). However, paired analysis revealed a significant decrease in baseline-to-follow-up LAP burden (*p* = 0.009; Figure 2C).

No significant differences were observed between the study arms in the baseline and follow-up LAP values (Table 3).

### 3.5. LAP Burden Reduction Predictors

**Figure 3:**
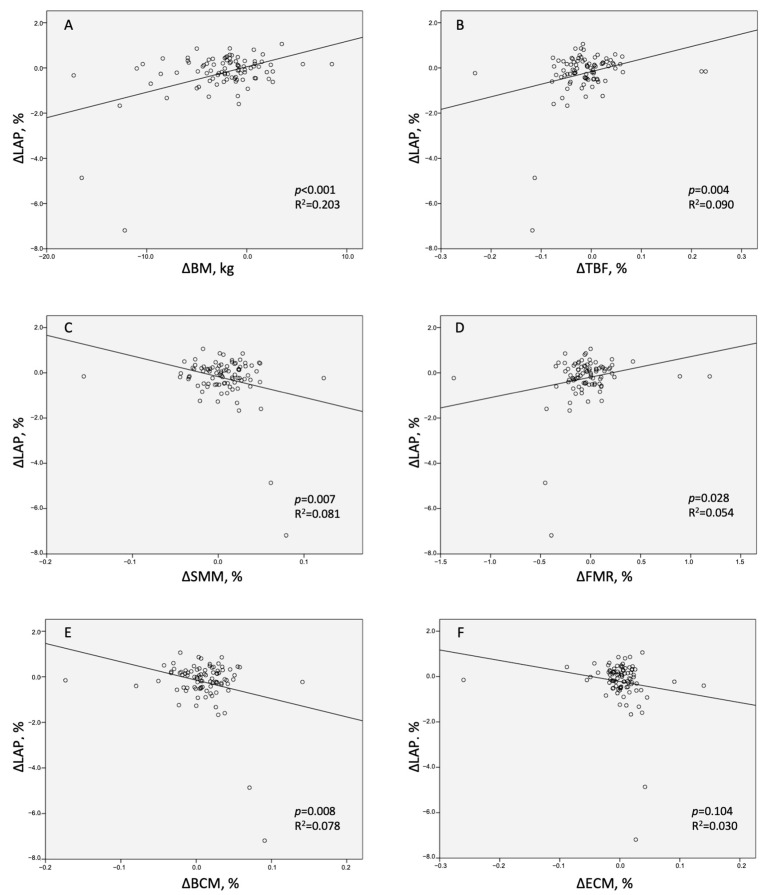
Scatter graphs presenting relationships between the change in low-attenuation coronary plaque (ΔLAP) and the changes in (**A**)—body mass (ΔBM), (**B**)—total body fat (ΔTBF), (**C**)—skeletal muscle mass (ΔSMM), (**D**)—fat-to-muscle ratio (ΔFMR), (**E**)—body cell mass (ΔBCM), and (**F**)—extracellular mass (ΔECM).

In the univariate linear regression model, a significant correlation was revealed between LAP reduction and BM reduction (Table 4 (Univariate Regression), Figure 3A). Regarding the individual body mass components, the correlation was positive for TBF and FMR (Table 4 (Univariate Regression), Figure 3B,D), and negative for SMM (Figure 3C). The multivariate linear regression model A identified BM as a predictor of LAP reduction independent of the study intervention arm, impaired glucose tolerance, and baseline body weight (Table 4 (Multivariate Regression Model A)). The multivariate linear regression model B identified TBF as the only component of body mass associated with LAP reduction, independently of the clinical cofactors (Table 4 (Multivariate Regression Model B)). The multivariate linear regression model C identified FMR as a predictor of LAP reduction independently of clinical cofactors (Table 4 (Multivariate Regression model C)).

## 4. Discussion

In this unique analysis, definite patterns of weight loss were identified as predictors of coronary plaque modification, that is, fat loss and decreased FMR were shown to be related to high-risk coronary plaque regression. These results underscore the importance of fat as the critical body mass component in the high-risk plaque reduction. Furthermore, our results may imply FMR as a plausible parameter to supervise anti-atherosclerotic weight loss/lifestyle interventions. To our knowledge, this is the first study to integrate serial coronary plaque analysis with body composition dynamics.

It is widely accepted that atherosclerosis and obesity share common pathophysiological features. Adipose tissue is currently referred to as a diffuse endocrine organ of complex metabolic activity [3,4,5,6]. Excessive fat accumulation leads to an imbalance between adipokines of pro- and anti-inflammatory potential. Obesity promotes the secretion of interleukin 1, interleukin 6, tumor necrosis factor alpha, and other pro-inflammatory factors that upregulate the IkappaB-kinase/nuclear factor kappa B (IKK/NF-κB) and mitogen-activated protein kinase (MAPK) pathways to induce cell apoptosis and ultimately contribute to myocardial injury [23,24]. Other mechanisms accounting for atherogenic activity of adipose tissue include the increase in fatty acid circulation, potentiating endothelial dysfunction, and oxidative stress [4]. Eventually, obesity-mediated inflammation contributes to nearly all stages of coronary plaque formation [5,6]. 

In contrast to adipose tissue, skeletal muscles mediate numerous mechanisms of anti-atherosclerotic effect. As a primary site for insulin-mediated glucose uptake, skeletal muscles are responsible for maintaining adequate insulin sensitivity. Higher muscle mass was shown to be associated with a lower risk of insulin resistance and prediabetes [25,26]. Interventions that increase muscle mass, such as cardiac rehabilitation and exercise training, can decrease insulin resistance [27,28] and may protect against endothelial dysfunction [29]. Preserved muscle mass provides a reliable protein reserve, which may reflect less muscle wasting secondary to circulating tumor necrosis factor alpha and interleukin 6 [7,10], and can be considered as an independent predictor of cardiovascular mortality, especially in older and/or chronically ill patients [30,31]. Sarcopenia, in turn, is associated with adverse glucose metabolism, and the phenomenon of progressive muscle wasting in patients with increased metabolic risk has been recognized as sarcopenic obesity, which is considered as a strong predictor of unfavorable outcomes [31,32]. Interestingly, a growing body of evidence is available on the metabolic activity of skeletal muscles by various cytokines and peptides that are produced, expressed, and released by muscle fibers. These bioactive substances, also called myokines, are involved in metabolic and cardiovascular regulation. Basic research data show that interleukin 15, myostatin, and irisin may exert cardioprotective effects [11,33,34]. The ability of myokines to balance and counteract the effects of adipokines has also been postulated [11].

Independent associations of fat mass and lean body mass on mortality in the general population have been postulated in the literature [18]. Studies involving body composition analysis showed that selective loss of fat mass was associated with reduced mortality [8], especially when accompanied by gain in fat-free mass [9] and, specifically, muscle mass [10]. It is noteworthy that high muscle mass accompanied by obesity may not guarantee a lower cardiovascular risk, and the need to balance between lean and fat mass should be further investigated in detail [35]. Our data add to these observations, with compelling evidence of inverse association between fat versus muscle percentage and high-risk coronary plaque progression.

This evidence should encourage concomitant monitoring of fat and muscle mass to predict cardiovascular outcomes. At this point, FMR emerges as a feasible parameter. Published data support the utility of FMR in metabolic risk stratification. Ramírez-Vélez et al. showed a significant association of high FMR and metabolic disorders in young Colombian adults [36]. Chen et al. and Xu et al. reported a similar association in patients with metabolic syndrome [37,38]. Furtherly, high FMR was pointed out as a predictor of insulin resistance and prevalence of T2DM [39,40,41]. Seo et al. showed a significant association of high FMR and insulin resistance in healthy Korean adults [39]. Wang et al. reported an increased risk of T2DM in patients with elevated FMR, independently of obesity measured with BMI [40]. In patients with treatment-naïve diabetes, FMR was postulated as a marker of liver fat accumulation [41]. In a study of diabetic patients performed by Liu et al., FMR was independently and positively associated with metabolic disorders, especially in women [42]. In this study, patients with a high FMR exhibited more cardiometabolic risk indicators, such as hypertension, fatty liver disease, abdominal obesity, insulin resistance, and adverse lipid profile.

Very importantly, two recently published longitudinal studies including nearly 500,000 UK Biobank participants followed-up for over 12 years revealed a positive association of FMR and both mortality [43] and incidence of CAD [44]. Our results may recognize a high-risk coronary plaque component as a mechanistic link responsible for these endpoints. The association of LAP and FMR demonstrates that the latter can serve as a plausible parameter reflecting changes in high-risk plaque burden. As a simple proportion of fat and muscle mass derived from noninvasive bioimpedance body composition analysis, FMR can be a clear, intelligible, and accessible parameter of importance in patients subjected to lifestyle-orientated interventions. Studies on larger groups are necessary to consolidate our findings and define the body composition goals associated with high-risk plaque reduction. In particular, it would be of interest to define the FMR threshold associated with LAP reduction below 4%, which was recently reported as the value related to lower cardiovascular outcomes [18]. In our study population, the median LAP percentage was substantially lower (about 1%) and, in fact, no cardiovascular events were observed during the follow-up [21].

Finally, our results emphasize the need for a comprehensive clinical surveillance to promote beneficial weight control patterns. When comparing weight reduction models in both study subgroups, pure fat reduction prevailed in the experimental arm (55% of weight reducers), while in the control arm, skeletal muscle reduction was most prevalent (34% of weight reducers, Figure 1B). Given a comparable percentage of combined fat and muscle reducers in both subgroups (25% and 29%), it may be hypothesized that nearly a third of weight reducers will lose fat rather than muscles when access to professional resources is provided, such as dietetic supervision and body composition analysis. Otherwise, intentional weight loss may not only be illusory, but actually deleterious, as a probable effect of muscle depletion.

Reversing adverse changes in body composition with diet and exercise regimens is feasible in patients with cardiovascular diseases [45]. Data show that body fat may be the compartment most susceptible to quantitative changes in response to diet and lifestyle interventions [46]. The recommendations of international cardiology associations underline the salutary role of moderate weight loss (5–10%) in the management of CAD [1] and consider weight loss of more than 5% as clinically relevant [2]. Our results suggest that even minor weight loss can result in a trend of vulnerable plaque regression, as long as desired proportions of body components are respected.

The small sample size and single-center study design are the main limitations of this study. In fact, the small sample size might result in the failure to demonstrate significant differences in ∆LAP between the study arms. Another limitation may be the variability of the quantitative plaque assessment method (QAngioCT); however, the software is approved and widely utilized in the leading imaging core labs and is considered one of the benchmarks for coronary plaque analysis. For this reason, we believe that the results are consistent. Undoubtedly, a blinded study protocol would substantially improve data quality; however, it is challenging for lifestyle-focused intervention trials to work out a blinded study design.

Since no previous data comparing body composition and LAP burden are available, further research is necessary to better interpret the obtained results. Further prospective studies on larger groups and more diversified patient populations, including diabetic patients, are necessary to confirm the findings of this pilot analysis and to search for an optimal body composition pattern for coronary plaque regression. Extending the laboratory panel with oxidative stress markers, specific chemokines, including adipo- and myokines, would provide valuable information for data interpretation. Also, using modern technologies such as physical activity monitoring devices or calorie counting applications might be a plausible option in future studies. Also, it would be of great interest to assess the sustainability of different weight loss patterns in an extended follow-up.

## 5. Conclusions

As opposed to routine management, intensive diet intervention resulted in a reduction in body mass associated with decreased fat and increased muscle mass. This weight reduction pattern was associated with a reduction in the low-attenuation coronary plaque.

Weight monitoring accounting for body mass components may provide insights into the dynamics of coronary plaque and allow us to recognize a weight reduction pattern associated with the decrease in the high-risk coronary plaque component. Compared to a simple weight control, tracking compartment-specific body composition changes over time can provide valuable information on adverse coronary plaque modification.

## Figures and Tables

**Table 1:** Patient clinical characteristics at baseline. SD—standard deviation; CCS—Canadian Cardiovascular Society (angina grading); BMI—body mass index; SBP—systolic blood pressure; DBP—diastolic blood pressure; HR—heart rate; BB—beta blocker; ACE-i—angiotensin-converting enzyme inhibitor; ARB—angiotensin receptor blocker; CCB—calcium channel blocker; IQR interquartile range; TC—total cholesterol; LDL—low-density lipoprotein; hs-CRP—high-sensitivity C-reactive protein.

	DISCO Intervention (*n* = 45)	Control (*n* = 44)	*p*-Value
Gender, female (%)	15 (33.3)	21 (47.7)	0.198
Age at baseline, years (SD)	59.4 (8.0)	60.6 (7.5)	0.468
Previous myocardial revascularization *, *n* (%)	3 (6.7)	1 (2.3)	0.616
Angina functional class, *n* (%)			
No angina or CCS 1	43 (95.6)	40 (90.9)	0.434
CCS 2	2 (4.4)	4 (9.1)	0.434
Prediabetes, *n* (%)	7 (11.1)	3 (6.8)	0.315
Dyslipidemia, *n* (%)	45 (100.0)	41 (93.2)	0.116
Hypertension, *n* (%)	41 (91.1)	38 (86.4)	0.522
Atrial fibrillation, *n* (%)	3 (6.7)	3 (6.8)	0.999
Smoking history, *n* (%)	27 (60)	27 (61.4)	0.999
Confirmed statin intolerance, *n* (%)	3 (6.7)	3 (6.8)	0.999
Overweight (BMI ≥ 25 kg/m^2^), *n* (%)	30 (84.4)	40 (90.9)	0.521
Obesity (BMI ≥ 30 kg/m^2^), *n* (%)	25 (55.5)	15 (34.1)	0.056
SBP, mmHg (SD)	129.7 (11.9)	129.7 (15.5)	0.999
DBP, mmHg (SD)	80.4 (6.5)	80.1 (8.2)	0.849
HR, min^−1^ (SD)	67.3 (7.2)	65.2 (13.6)	0.368
Antiplatelet treatment, *n* (%)	31 (68.9)	26 (59.0)	0.335
Statin treatment, *n* (%)	29 (64.4)	31 (70.5)	0.652
BB treatment, *n* (%)	24 (53.3)	28 (62.2)	0.391
ACE-IARB treatment, *n* (%)	30 (66.6)	34 (77.3)	0.347
CCB treatment, *n* (%)	15 (33.3)	13 (29.5)	0.808
Diuretic treatment, *n* (%)	12 (26.7)	17 (38.6)	0.263
Median number of antihypertensive drugs (IQR)	2.0 (1.0–3.0)	2.0 (1.25–3.0)	0.380
Baseline TC, mg/dL (SD)	181.9 (44.4)	177.6 (42.8)	0.643
Baseline LDL, mg/dL (SD)	108.6 (37.9)	109.6 (39.6)	0.905
Baseline hs-CRP, mg/L	0.13 (0.09, 0.30)	0.14 (0.09, 0.22)	0.671
Baseline homocysteine, umol/L (SD)	13.2 (4.3)	14.1 (8.7)	0.540
Observation time, weeks (SD)	69.4 (15.8)	64.3 (10.7)	0.079

* Percutaneous coronary angioplasty only (no coronary artery bypass grafting).

**Table 2:** Body mass and body composition measurements at baseline, follow-up, and changes (∆) throughout the study observation. BM—body mass; BMI—body mass index; TBF—total body fat; FFM—fat-free mass; BCM—body cell mass; SMM—skeletal muscle mass; TBW—total body water; CI—confidence interval.

	Study Arm							
	DISCO Intervention (*n* = 45)			Control (*n* = 44)			*p*-Value	95% CI
	Min	Max	Mean ± SD	Min	Max	Mean ± SD		
BM baseline, kg	45.9	116.6	84.9 ± 16.2	51.6	121.9	82.2 ± 14.9	0.424	−3.9; 9.2
BM follow-up, kg	53.0	107.0	81.3 ± 13.8	50.7	117.9	80.8 ± 14.9	0.862	−5.5; 6.6
∆BM, kg	−17.3	8.5	−3.6 ± 4.9	−8.4	5.6	−1.4 ± 2.9	**0.015**	−3.8; −0.4
BMI baseline, kg/m^2^	17.8	36.5	29.8 ± 4.2	21.2	40.2	29.1 ± 3.8	0.431	−1.0; 2.4
BMI follow-up, kg/m^2^	21.1	35.5	28.6 ± 3.6	20.8	38.9	28.6 ± 3.8	0.989	−1.6; 1.5
∆BMI, kg/m^2^	−5.4	3.3	−1.2 ± 1.6	−3.4	1.7	−0.5 ± 1.1	**0.020**	−1.3; −0.1
TBF baseline, kg	7.3	58.6	28.7 ± 9.8	14.9	48.9	26.9 ± 7.8	0.323	−1.9; 5.6
TBF follow-up, kg	9.3	45.7	24.5 ± 7.7	13.1	43.3	27.1 ± 7.8	0.119	−5.8; 0.7
∆TBF, kg	−24.8	4.9	−4.2 ± 5.3	−8.8	15.7	0.2 ± 4.2	**<0.001**	−6.5; −2.4
FFM baseline, kg	34.7	80.2	56.2 ± 11.8	36.7	75.5	55.3 ± 12.1	0.758	−4.3; 5.8
FFM follow-up, kg	35.8	75.1	56.8 ± 11.3	23.8	74.6	53.7 ± 13.2	0.235	−2.1; 8.3
∆FFM, kg	−7.1	22.0	0.6 ± 4.4	−16.7	3.5	−1.6 ± 3.9	**0.010**	0.6; 4.1
BCM baseline, kg	22.0	52.2	36.5 ± 7.7	23.6	49.3	35.9 ± 8.0	0.734	−2.7; 3.9
BCM follow-up, kg	22.8	48.3	36.7 ± 7.1	21.9	48.3	35.3 ± 8.2	0.403	−1.9; 4.5
∆BCM, kg	−7.9	13.3	0.2 ± 3.0	−10.1	2.5	−0.6 ± 2.2	0.156	−0.3; 1.9
SMM baseline, kg	18.1	45.5	31.2 ± 7.0	19.5	42.9	30.7 ± 7.3	0.734	−2.5; 3.5
SMM follow-up, kg	18.8	42.0	31.6 ± 6.6	17.9	42.0	29.7 ± 7.6	0.211	−1.1; 4.9
∆SMM, kg	−4.3	11.6	0.4 ± 2.6	−9.2	2.1	−1.0 ± 2.1	**0.006**	0.4; 2.4
TBW baseline, kg	25.3 (*n* = 39)	53.9 (*n* = 39)	40.9 ± 8.3	26.8	55.1	40.2 ± 8.8	0.375	−4.4; 3.1
TBW follow-up, kg	26.1	55.0	41.2 ± 8.1	24.6	53.8	39.3 ± 8.8	0.296	−5.4; 1.7
∆TBW, kg	−5.7 (*n* = 39)	6.6 (*n* = 39)	0.3 ± 2.2	−13.1	3.6	−0.9 ± 3.1	**0.040**	−2.4; −0.1

*p*-Values < 0.005 were marked in bold.

**Table 3:** Changes in body mass, body mass components and low-attenuation coronary plaque burden between the study arms; *p*-values <0.005 are marked in bold; ∆—change (delta); BM—body mass; TBF—total body fat; BCM—body cell mass; ECM—extracellular mass; SMM—skeletal muscle mass; FMR—fat-to-muscle ratio; LAP—low-attenuation plaque; CI—confidence interval; * other than normally distributed data.

		Study Arm		*p*-Value	95% CI Z-Score *
		DISCO Intervention (*n* = 45)	Control (*n* = 44)		
Body Mass	BM baseline, kg	84.9 ± 16.2	82.2 ± 14.9	0.424	−3.90; 9.22
	BM follow-up, kg	81.3 ± 13.8	80.8 ± 14.9	0.862	−5.51; 6.58
	∆BM, kg	−3.6 ± 4.9	−1.4 ± 2.9	**0.015**	−3.83; −0.42
Three-Compartment Body Composition Model	TBF baseline, %	33.5 ± 8.7	32.8 ± 7.6	0.688	−0.03; 0.04
	TBF follow-up, %	30.1 ± 8.0	33.9 ± 9.2	**0.040**	−0.07; −0.01
	∆TBF, %	−3.4 ± 4.8	1.1 ± 5.5	**<0.001**	−0.1; −0.02
	BCM baseline, %	43.2 ± 5.6	43.6 ± 5.0	0.738	−0.03; 0.02
	BCM follow-up, %	45.2 ± 5.4	43.5 ± 5.5	0.133	−0.01; 0.04
	∆BCM, %	2.0 ± 3.3	−0.1 ± 3.7	**0.007**	0.01; 0.04
	ECM baseline, %	23.3 ± 3.2	23.6 ± 3.1	0.638	−0.02; 0.01
	ECM follow-up, %	24.7 ± 3.8	22.6 ± 1.5	**0.030**	0.01; 0.04
	∆ECM, %	1.4 ± 3.0	−1.0 ± 4.3	**0.003**	0.01; 0.04
Skeletal Muscles and Fat-To-Muscle Mass Ratio	SMM baseline, %	36.9 ± 5.0	37.2 ± 4.6	0.779	−0.02; 0.02
	SMM follow-up, %	38.8 ± 4.8	36.5 ± 5.3	**0.032**	0.01; 0.04
	∆SMM, %	1.9 ± 2.8	−0.7 ± 3.2	**<0.001**	0.01; 0.04
	FMR baseline, %	88.5 (65.5; 115.8)	88.3 (62.9; 111.5)	0.718	−0.361
	FMR follow-up, %	76.9 (56.3; 94.1)	93.5 (70.2; 120.2)	**0.046**	−1.994
	∆FMR, %	−12.9 (−21.2; −4.3)	3.1 (−5.3; 10.7)	**<0.001**	−4.801
High-Risk Coronary Plaque	LAP baseline, %	1.26 (0.94; 2.03)	1.25 (0.94; 1.63)	0.555	−0.591
	LAP follow-up, %	1.07 (0.94; 1.74)	1.23 (0.89; 1.67)	0.948	−0.66
	∆LAP, %	−0.11 (−0.61; 0.28)	0.06 (−0.39; 0.23)	0.286	−1.067

**Table 4:** Linear regression: 1. univariate, 2–4. multivariate stepwise regression models showing relationships between body mass body components, clinical characteristics, and low-attenuation plaque (LAP). *p*-values < 0.05 are marked in bold, and *p*-values 0.05–0.01 are marked with asterisks (*); ∆—change (delta); BM—body mass; TBF—total body fat; BCM—body cell mass; ECM—extracellular mass; SMM—skeletal muscle mass; FMR—fat-to-muscle ratio; BMI—body mass index; TC—total cholesterol, LDL—low-density lipoproteins; hs-CRP—high sensitivity C-reactive protein; CI—confidence interval.

Title 1	Title 2	*p*-Value	Unstandardized B	Standardized B	95% CI
1. Univariate Regression	∆BM	**<0.001**	0.113	0.450	0.065; 0.160
	∆TBF	**0.004**	5.564	0.300	1.792; 9.335
	∆BCM	**0.008**	−8.065	−0.279	−13.978; −2.153
	∆ECM	0.104	−4.641	−0.174	−10.248; 0.967
	∆SMM	**0.007**	−9.156	−0.285	−15.7811; −2.601
	∆FMR	**0.028**	0.908	0.233	0.100; 1.716
	∆BMI	**<0.001**	0.321	0.436	0.180; 0.462
	Age	0.587	0.008	0.058	−0.021; 0.037
	Sex	0.170	0.311	0.147	−0.135; 0.756
	Study arm	0.076 *	−0.393	−0.189	−0.827; 0.042
	Arterial hypertension	0.520	−0.227	−0.069	−0.926; 0.472
	Hyperlipidemia	0.336	−0.593	−0.103	−1.812; 0.626
	Statin treatment	0.615	0.137	0.054	−0.402; 0.676
	Antiplatelet treatment	0.641	−0.119	−0.050	−0.624; 0.386
	Atrial fibrillation	0.969	−0.016	−0.004	−0.838; 0.806
	Baseline TC	0.671	0.001	0.046	−0.004; 0.006
	Baseline LDL	0.832	0.001	0.023	−0.005; 0.006
	Baseline hs-CRP	0.360	0.520	0.100	−0.604; 1.643
	Baseline homocysteine	0.946	0.001	0.007	−0.032; 0.034
	Smoking history	0.442	−0.223	0.083	−0.797; 0.351
	Impaired glucose tolerance	**0.031**	−0.833	−0.229	−1.585; −0.080
	Prior coronary revascularization	0.905	−0.065	−0.013	−1.132; 1.003
	Baseline BM	0.090 *	−0.012	−0.181	−0.026; 0.002
	Baseline BMI	0.116	−0.043	−0.168	−0.098; 0.011
	Obesity (BMI ≥ 30 kg/m^2^)	0.579	−0.124	−0.060	−0.568; 0.320
	Overweight (BMI ≥ 25 kg/m^2^)	0.644	−0.157	−0.050	−0.828; 0.514
	Baseline calcium score	0.171	−0.001	−0.147	−0.001; 0.001
2. Multivariate Regression Model A ^1^	∆BM	**<0.001**	0.113	0.450	0.065; 0.160
	Study arm	0.427	-	−0.079	-
	Impaired glucose tolerance	0.156	-	−0.139	-
	Baseline BM	0.874	-	0.017	-
3. Multivariate Regression Model B ^2^	∆TBF	**0.004**	5.564	0.300	1.792; 9.335
	∆BCM	0.383	-	−0.130	-
	∆ECM	0.383	-	0.141	-
	Study arm	0.467	-	−0.082	-
	Impaired glucose tolerance	0.061	-	−0.193	-
	Baseline BM	0.340	-	−0.102	-
4. Multivariate Regression model C ^3^	∆FMR	**0.028**	0.908	0.233	0.100; 1.716
	Study arm	0.312	-	−0.115	-
	Impaired glucose tolerance	0.050	-	−0.205	-
	Baseline BM	0.233	-	0.233	-

^1^ R^2^ = 0.203; ANOVA F = 22.135; *p* < 0.001; ^2^ R^2^ = 0.090; ANOVA F = 8.595; *p* = 0.004); ^3^ R^2^ = 0.054; ANOVA F = 4.992; *p* = 0.028.

## Data Availability

The data that support the findings this study are available from the corresponding author upon reasonable request.

## Appendix A

Inclusion and exclusion criteria of Dietary Intervention to Stop Coronary Atherosclerosis in Computed Tomography (DISCO-CT) Trial (NCT02571803) are presented in Table A1.

**Table A1:** Inclusion and exclusion criteria of Dietary Intervention to Stop Coronary Atherosclerosis in Computed Tomography (DISCO-CT) Trial. CCTA—coronary computed tomography angiography; ACC—American College of Cardiology; AHA—American Heart Association; CABG—coronary artery bypass grafting.

Inclusion Criteria
Coronary atherosclerosis lesions confirmed in the CCTA defined as maximum luminal stenosis < 70% in at least 2 coronary artery segments (according to ACC/AHA classification)
No indications for coronary angiography/revascularization (no documented significant ischemia of the myocardium)
Age > 18 years
Informed consent to participate in the study
Declaration and willingness to cooperate throughout the study
Coronary atherosclerosis lesions confirmed in the CCTA defined as maximum luminal stenosis < 70% in at least 2 coronary artery segments (according to ACC/AHA classification)
**Exclusion Criteria**
Valvular heart disease or other known condition requiring cardiac surgery (or expected cardiac surgery intervention) within 12 months
Dilated or hypertrophic cardiomyopathy
Diabetes mellitus type 2
Past CABG procedure
Known genetic disorders affecting the development of atherosclerotic lesions (e.g., familial hyperlipidemia, congenital metabolic disorders)
Factors that may affect the quality and safety of CCTA examination (e.g., atrial fibrillation, significant ventricular arrhythmias, poor patient cooperation, renal insufficiency, women at childbearing age) or low quality of data obtained from CCTA

## Appendix B

The Dietary Intervention to Stop Atherosclerosis in Computed Tomography (DISCO-CT) study design flow chart is presented in Figure A1.
